# Potential influence of automated volumetry on treatment response classifications in lung cancer lesions

**DOI:** 10.1186/1470-7330-14-S1-P30

**Published:** 2014-10-09

**Authors:** Kaveh Akbari, Alexandra Barthol, Irene Schnauder, v Wunn, Elmar Brehm, Bernd Lamprecht, Franz Fellner

**Affiliations:** 1 Department of Radiology, General Hospital Linz, Linz, Austria

## Purpose

To evaluate the potential influence of automated volumetry on treatment response classifications in lung cancer in comparison to manual unidimensional measurements.

## Material and methods

60 patients (41 men, 19 women, mean age 61.8 ± 9.4 years) with histopathologically verified lung cancer were included in this retrospective study.

For each patient, up to 2 target lesions were quantitatively evaluated in a baseline and two follow-up CT scans (77 lesions, 154 response classifications) by two independent radiologists. Hilar and mediastinal masses, as well as lesions surrounded by atelectasis were excluded.

For each lesion a unidimensional diameter measurement, as well as an automated CT-volumetry was performed. In the follow-up studies, the response evaluation was assessed using RECIST compared to volume equivalents of RECIST with converted thresholds (-65/+73%).

## Results

The results of the manual one-dimensional measurements varied between the two observers by 6.34 ± 17.12%, affecting the volume to the power of 3, whereas the volumetric measurements varied only by 3.33 ± 6.66%.

In 16.9% (26/154) of the cases the volumetric assessment led to a different response classification.

In 13% (20/154) of the cases the different response classification would have an effect on therapeutic decisions.

## Conclusion

The volumetric assessment of lung cancer lesions can reflect the tumour burden more appropriately and therefore has a significant effect on response classifications and therapeutic decisions.

